# Monogonont Rotifer, *Brachionus calyciflorus*, Possesses Exceptionally Large, Fragmented Mitogenome

**DOI:** 10.1371/journal.pone.0168263

**Published:** 2016-12-13

**Authors:** Zhi-Juan Nie, Ruo-Bo Gu, Fu-Kuan Du, Nai-Lin Shao, Pao Xu, Gang-Chun Xu

**Affiliations:** Key Laboratory of Freshwater Fisheries and Germplasm Resources Utilization, Ministry of Agriculture, Freshwater Fisheries Research Center, Chinese Academy of Fishery Sciences, Wuxi, China; Sichuan University, CHINA

## Abstract

In contrast to the highly conserved mitogenomic structure and organisation in most animals (including rotifers), the two previously sequenced monogonont rotifer mitogenomes were fragmented into two chromosomes similar in size, each of which possessed one major non-coding region (mNCR) of about 4–5 Kbp. To further explore this phenomenon, we have sequenced and analysed the mitogenome of one of the most studied monogonont rotifers, *Brachionus calyciflorus*. It is also composed of two circular chromosomes, but the chromosome-I is extremely large (27 535 bp; 3 mNCRs), whereas the chromosome-II is relatively small (9 833 bp; 1 mNCR). With the total size of 37 368 bp, it is one of the largest metazoan mitogenomes ever reported. In comparison to other monogononts, gene distribution between the two chromosomes and gene order are different and the number of mNCRs is doubled. *Atp8* was not found (common in rotifers), and *Cytb* was present in two copies (the first report in rotifers). A high number (99) of SNPs indicates fast evolution of the *Cytb-1* copy. The four mNCRs (5.3–5.5 Kb) were relatively similar. Publication of this sequence shall contribute to the understanding of the evolutionary history of the unique mitogenomic organisation in this group of rotifers.

## Introduction

Metazoan mitogenomes are one of the most conservative genomic regions in terms of gene content and organization: commonly a single, closed, circular molecule ranging from about 15 to 20 kb, containing 37 genes (13 protein-coding, 2 rRNAs, and 22 tRNAs). Metazoan mtDNA is usually very compact: there are generally no introns, little or no intergenic DNA, and most genes show clear signatures of selection for small size [1,2]. Nevertheless, fragmentation of the “standard” single-circle mtDNA into multichromosomal genomes, which is common in higher plants [3,4], has occurred independently in some metazoans as well: nematodes [5], mesozoans [6], cnidaria [7], insects [8], and some monogonont rotifers [9,10].

Rotifers are a model system for studies of the evolution of sex [11], as well as aquatic food webs, predator-prey dynamics, population dynamics, speciation, ecotoxicology and evolutionary adaptation [12,13]. The taxonomy of Syndermata (or Rotifera *sensu lato*) is still unresolved, but the clade is usually divided into Seisonidea, Acanthocephala, Bdelloidea and Monogononta classes [14–16]. Monogononta are cosmopolitan, predominantly freshwater, rotifers that normally reproduce by cyclical parthenogenesis (an alternation between ameiotic parthenogenesis and sporadic sexual episodes), although some populations lose the ability to reproduce sexually [17]. In 2008, the sequencing of the first complete mitogenome of a monogonont rotifer, *Brachionus plicatilis*, has revealed a unique structure: two circular chromosomes (about 11 and 12.5 Kbp), both possessing a long (mostly) non-coding region of about 4.9 Kbp [9]. Only one monogonont mitogenome, *B*. *koreanus*, has been published subsequently, and it was revealed to be structurally very similar to *B*. *plicatilis* [10]. *B*. *rubens* mitogenome is also available from the GenBank (KJ489417 and KJ489418), but currently (Nov, 2016) remains unpublished. Mitogenome sequence of one of the most abundant and most studied monogonont rotifers, *Brachionus calyciflorus*, is currently not available. It is a cryptic species complex, occurring in freshwater ecosystems, that represents an excellent system for inferring the evolutionary processes of phylogeographic structure due to its patchy nature of habitats, lack of hybridization and cyclical parthenogenesis [18,19]. In order to further explore the phenomenon of fragmented mitogenomes in monogonont rotifers, we have sequenced and analysed the mitogenome of *Brachionus calyciflorus*.

## Materials and Methods

### Samples and DNA extraction

Plankton sample was collected using plankton nets (64 microns) from an outdoor pond (N 31°25′46.45”, E 120°16’54.38”) at the Freshwater Fisheries Research Center of the Chinese Fisheries Science Research Institute South. As this is an experimental pond belonging to our institute, and the field studies did not involve endangered or protected species, no permissions were required for sampling. Samples were collected in a 1 L beaker, filtered using a 165 micron mesh to remove large cladocerans, copepods and organic waste, and washed 2–3 times. A single rotifer was chosen under a microscope using a pipette, morphologically identified as *Brachionus calyciflorus* [20,21] and cultured at 26°C in an incubator with artificial illumination. Culture medium (100 mL) contained the water from the pond (55 micron mesh-filtered and autoclaved), 0.1 mL/L of a custom-made composite bacterial broth (probiotics, *Bacillus* spp. and *Lactobacillus* spp., and sterilised commercial fermented organic fertiliser) and 0.06 mL/L fresh *Chlorella* spp. (cultivated in a standard BG-11 medium to OD680≈0.8). In approximately 7 to 10 days, when the number of individual rotifers in the population reached about 100 (1 rotifer/mL, log phase), rotifers were mesh-filtered again (55 microns), checked for the presence of other animal species under a microscope, and moved into a fresh medium (200 mL). This batch (No. 2) was cultured until the concentration reached about 4 rotifers/mL (log phase), rotifers again collected as described, placed in a fresh 500 mL medium (batch No. 3), and further cultured until the concentration reached about 10 rotifers/mL (log phase). At the end of this step, rotifers were harvested using plankton net (55 micron mesh). To minimize the amount of *Chlorella* in the sample, we have rinsed the collected rotifers with distilled water several times and then placed them in a fresh distilled water medium for 24h to allow them to digest and excrete any remaining algae. Eventually, rotifers were harvested again, placed in 100% ethanol and preserved at -20°C. About 150 mg (wet weight) of rotifers was obtained for the DNA extraction.

### PCR amplification, sequencing and annotation

DNA was extracted using Aidlab DNA extraction kit (Aidlab Biotechnologies, Beijing), and *18S* and *Cox1* fragments amplified (Table 1) and sequenced to corroborate the accuracy of the morphological identification [16,18,22]. Degenerate primers were designed to match the generally conserved regions of mtDNA genes and used to amplify and sequence short fragments of *12s*, *Cox1*, *Cox3*, *Cytb*, *Nad1* and *Nad4-5* genes. Specific primers were then designed based on these sequences and used to amplify long-chain products in several PCR reactions. These primers were designed to amplify products with overlaps of about 100 bp. Reaction volume of 50 μL contained 1.0 μL of LongAmp® *Taq* polymerase (New England BioLabs, Inc.), 10 μL of 5x LongAmp® Taq Reaction Buffer, 1.0 μL dNTP mix (10 mM), 5.0 μL DNA template, 1.5 μL each primer (10x), and 30μL PCR-grade H_2_O. PCR conditions were optimized for each reaction, with the annealing temperature adjusted to suite the specific primer pair, extension time set to 1 min per Kb of expected product size, and cycles (35 on average) adjusted depending upon the amplification efficiency of the primers. PCR products were sequenced directly by the dideoxynucleotide procedure, using an ABI 3730 automatic sequencer. When that was not possible, the products were cloned into a pMD18-T vector (TaKaRa) and then sequenced. Sequences were assembled in a stepwise manner, ensuring that the overlaps are identical, hence confirming that no numts or fragments of *Chlorella* DNA were mistakenly incorporated during the mitogenome sequence assembly. All obtained fragments were BLASTed [23] to confirm that the amplicon is the actual target sequence. *DNAstar v7*.*1* (Dnastar Inc., USA) was used to assemble the sequences and locate the putative ORFs for protein-coding genes (PCGs). Then we used *BLAST* and *Blastx* to compare the putative ORFs with published nucleotide and amino acid sequences of related species, and manually determine the actual initiation codon and termination codon positions. Annotation of tRNAs was performed using *tRNAscan* [24] and *ARWEN* [25] and the results checked manually. *MEGA7* [26] was used for similarity analyses and to conduct sequence alignments and OGDRAW to visualize the genome [27].

**Table 1:** Primers used for PCR amplification of the *Brachionus calyciflorus* mitochondrial genome.

Primer	Sequence (5’-3’)	Gene/size
**chromosome-I**		
LCCYTBF	GTACTYCCTWGAGRTCAADTGTC	*Cytb-1*
LCCYTBR	CCTARTTYATTAGRAATAGAKCG	473
1LCF1	TGCAGAGAATTTTATTCCTGC	*Cytb-12S*
1LCR1	TGTATGATGGTGGATCATCC	6478
LC12SF	AAGAMAAGGWTTAGATAYCC	*12S*
LC12SR	CADAGGTGWCGGGYGATTTGT	367
1LCF2	GCTTAATTCGGATTTGAAAG	*12S-Nad1*
1LCR2	ATGTGTCTCTGCTAAAGTAC	1860
LCND1F	TAGYGCAGWCTATTTDTTACGA	*Nad1*
LCND1R	AATCYTGACWCTAGTYCAGACTC	211
1LCF3	GATCTTTTAGGATGTCTTCAC	*Nad1-16S*
1LCR4	GAGCTAGTTAGAATTTTCAC	1943
LC16SF	GTACYCTGADTGTGCTRAGGYAGC	*16S*
LC16SR	CGGTTTRAADTCAGAYCATRTA	411
1LCF5	ATTACTACCTCGATGTTGG	*16S-Cox3*
2LCR4	GAAAAGTGGTGGTTATACAG	7693
LCCOX3F	ACARGATTCCWTGGTAYACAKGT	*Cox3*
LCCOX3R	CAGAYAACAWCTACGARGTRTCA	146
2LCF5	GTAGGTACTATTTTTTTATTCTATG	*Cox3- Cox1*
2LCR7	TCTCTTTGTTGTACGAGAAC	7611
LCCOX1F	ACCRGTTGAAYTGTGTWTCCTCC	*Cox1*
LCCOX1R	GCTDCAAYATAAHAAGYATKATG	761
2LCF1	CTTCAGTACCTTTACTTTGAG	*Cox1- Cytb2*
1LCR5	CGAGTTAAAGTAGGATTACC	1217
**chromosome-II**		
LCND4F	CCTHTAGYTCATGWGGRGGMTCC	*Nad4-Nad5*
LCND5R	GGAYTTGDATAACRCATVAGT	1676
2LCF3.1	CTTCAACTCTAGTTACAGCAGGC	*Nad5- Nad3*
LCDoR	CATATATTGGTATAGGGGTTG	1859
2LCF3.4	CTTTAGAGTGACTATCTTGG	*Nad3- Nad6*
2LCR1.6	GCAGATTGCACAAGTATAACCA	5873
2LCF1-7	ATGACAATCTTATTAAGCTT	*Nad6-Nad4*
2LCR1.1	CAAACAACAGTAACATAAACTAAG	1496
***18S***		
LC18SF	ACCTGGTTGATCCTGCCAG	*18S*
LC18SR	GATCCTTCTGCAGGTTCAC	1800

## Results and Discussion

### Genome size and organisation

**Fig 1:**
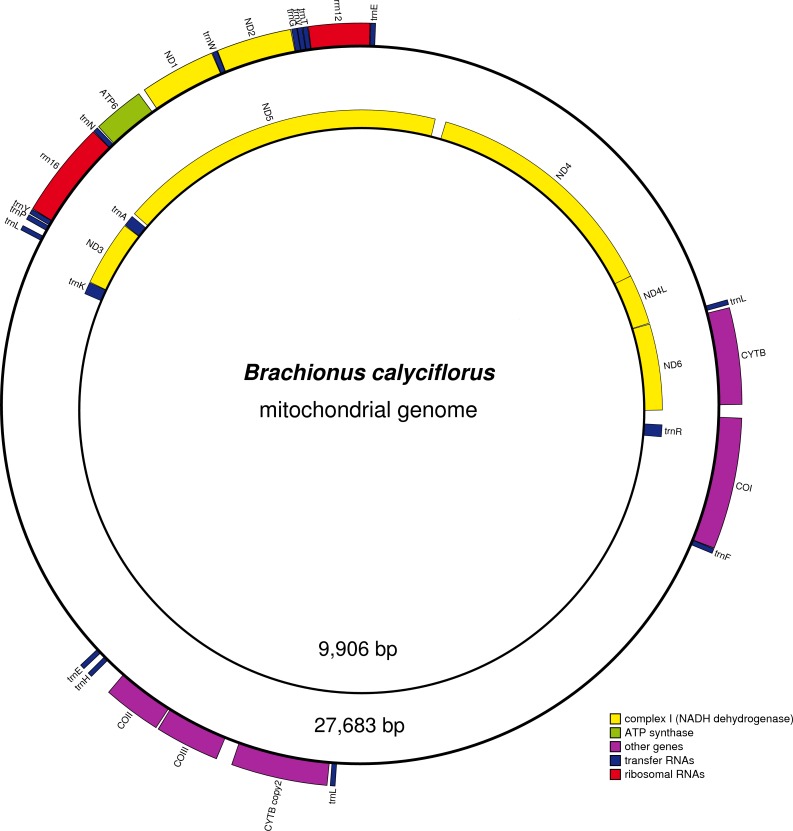
Maps of the two *B*. *calyciflorus* mitochondrial chromosomes.

The mitogenome of *B*. *calyciflorus* is, just as the other three available monogonont mitogenomes, also composed of two circular chromosomes. However, chromosome-I (mtDNA-I, GenBank accession number KX822781) is extremely large—27 535 bp, whereas chromosome-II (mtDNA-II, KX822782) is smaller than in other monogononts—9 833 bp (Fig 1). The corresponding sizes in other monogononts are: 10 421 / 11 923 bp in *B*. *koreanus* [10], 11 153 / 12 672 bp in *B*. *plicatilis* [9], and 11 398 / 12 820 in *B*. *rubens*. With the total size of 37 368 bp, *B*. *calyciflorus* mitogenome is among the largest ever reported in metazoans [28]; the only metazoan mitogenomes comparable in size were found in some molluscs: 30 to 40 Kb in *Placopecten magellanicus* [29] and 47 to 50 Kb in *Scapharca broughtonii* [30]. Variation in the length of non-coding regions (NCRs) generally accounts for much of the size variability in mitogenomes, mostly due to segmental duplications [28]. For example, there appears to be no functional constraint on the size of the intergenic regions in the angiosperm mitogenomes, which are thus the largest and least gene-dense eukaryotic mitochondrial genomes [4]. This is also the case in *B*. *calyciflorus* mitogenome, where the exceptional size is a result of the existence of four major NCRs (mNCRs): one in the chromosome-II (5441 bp), and three in the chromosome-I (5386, 5391 and 5369 bp). We have not studied whether the size of the chromosomes varies among individual specimens, but the chromosome size and gene content among *B*. *plicatilis* individuals were relatively constant [9].

Whereas the gene order in vertebrates is almost invariant, in invertebrates it is relatively variable. However, this is mostly due to the variation in number and location of tRNAs, whereas the order of PCGs and rRNAs is very stable [29]. The gene order in *B*. *koreanus* and *B*. *plicatilis* was almost identical, save for one rearrangement between tRNA^Arg^ and tRNA^Ile^ [10]. However, *B*. *calyciflorus* mitogenome is an outlier among the monogonont mitogenomes in terms of gene order as well. Furthermore, even the gene content on the two chromosomes was notably different from the remaining three available monogonont mitogenomes. Whereas *Cytb*, *12s*, *Nad2*, *Nad1*, *Atp6* and *16s* genes are present (in that order) on the first chromosome of all four monogonont genomes, *B*. *calyciflorus* chromosome-I also contains *Cox2*, *Cox3*, an extra copy of *Cytb* and *Cox1* (Table 2, Fig 1). Gene content (tRNAs notwithstanding) and order on chromosome-II are also almost identical in the remaining three mitogenomes: *Cox1*, *Nad6*, *Nad4l*, *Nad5*, *Cox2*, *Cox3* and *Nad3*. In *B*. *calyciflorus*, however, it contains only *Nad6*, *Nad4l*, *Nad4*, *Nad5* and *Nad3* (in that order). Gene order (including the PCGs) is also very different from the one previously reported in an Acanthocephalan, *Seison* sp. [15].

**Table 2:** Structural features and organisation of the *Brachionus calyciflorus* mitochondrial genome.

No.	Gene	Position		Size	IGN	Codon		Anti-
		Start	End	bp	bp	Start	Stop	Codon
**chromosome-I**								
1	*Cytb-1*	1	1140	1140		ATG	TAA	
2	*tRNA*^*Leu-1*^	1169	1232	64	28			TAG
3	mNCR-1	1233	6618	5386				
4	*tRNA*^*Glu-1*^	6739	6803	65	120			TTC
5	*12S rRNA*	6804	7525	722				
6	*tRNA*^*Thr*^	7526	7589	64				TGT
7	*tRNA*^*Val*^	7590	7653	64				TAC
8	*tRNA*^*Gly*^	7652	7719	68	-2			TCC
9	*Nad2*	7723	8613	891	3	ATG	TAA	
10	*tRNA*^*Trp*^	8612	8675	64	-2			TCA
11	*Nad1*	8676	9573	898		ATG	T—	
12	*tRNA*^*Gln*^	9575	9639	65	1			TTG
13	*Atp6*	9640	10251	612		ATG	TAA	
14	*tRNA*^*Asp*^	10262	10325	64	10			GTC
15	*16S rRNA*	10326	11469	1144				
16	*tRNA*^*Tyr*^	11470	11532	63				GTA
17	*tRNA*^*Pro*^	11542	11608	67	9			TGG
18	*tRNA*^*Met*^	11609	11674	66				CAT
19	*tRNA*^*Leu-2*^	11684	11747	64	9			TAA
20	mNCR-2	11748	17138	5391				
21	*tRNA*^*Glu-2*^	17139	17203	65				TTC
22	*tRNA*^*His*^	17266	17328	63	62			GTG
23	*Cox2*	17587	18282	696	258	GTG	TAA	
24	*Cox3*	18302	19066	765	19	ATA	TAA	
25	*tRNA*^*Cys*^	19097	19157	61	30			GCA
26	*Cytb-2*	19245	20384	1140	87	ATG	TAA	
27	*tRNA*^*Leu-3*^	20413	20476	64	28			TAG
28	mNCR-3	20477	25845	5369				
29	*tRNA*^*Phe*^	25846	25909	64	75			GAA
30	*tRNA*^*Ser*^	25910	25974	65				TGA
31	*Cox1*	25985	27535	1551	10	ATG	TAA	
**chromosome-II**								
1	*Nad6*	1	462	462		ATG	TAG	
2	*Nad4L*	464	733	270	1	ATG	TAA	
3	*Nad4*	730	2031	1302	-4	ATA	TAA	
4	*Nad5*	2085	3777	1693	53	ATG	TAA	
5	*tRNA*^*Asn*^	3778	3841	64				GTT
6	*tRNA*^*Ala*^	3845	3906	62	3			TGC
7	*Nad3*	3907	4258	352		ATG	T—	
8	*tRNA*^*Lys*^	4259	4325	67				TTT
9	mNCR-1	4326	9766	5441				
10	*tRNA*^*Arg*^	9767	9833	67				ACG
11	*tRNA*^*Ile*^	9842	9906	65	8			GAT

IGN is the number of inter-genetic nucleotides, where a positive value indicates a non-coding region, whereas a negative value indicates a gene overlap.

### Genome characteristics

Despite the unusually large size and unusual organisation, the mitogenome contained most of the standard 37 mtDNA genes. Only *Atp8* was not found among the standard 13 mitochondrial protein-coding genes, but *Cytb* was present in two copies. The absence of *Atp8* is common in rotifers [15,28,31]. Duplicated protein coding genes are common in mitogenomes of some metazoan groups [32], but with the exception of mitogenomes of some birds, characterized by a tandemly duplicated region encompassing a part of the *Cytb*, three *tRNAs*, *Nad6* and the control region [33], the duplication of *Cytb* appears to be a relatively rare event in metazoans. Even though the two *Cytb* copies were very similar (about 99.1%), a high number (99) of SNPs indicates a relatively fast evolution of one of the copies, probably facilitated by the functional redundancy. Similarity and phylogeny analyses including available orthologs (not shown) indicate that *Cytb-1* is the faster-evolving copy.

Apart from the already discussed genes, chromosome-I and II also contained 18 and five tRNAs, respectively. Generally, tRNAs are the gene category with the highest ‘dispensability’ in the mitochondrial genome [28], but all of the main 20 tRNAs were found in the *B*. *calyciflorus* mitogenome. However, only 18 were found using the available annotation programs: 15 on the chromosome-I and three on the chromosome-II. Only 15 of these were unique: tRNA^Glu^ was found in two and tRNA^Leu^ in three copies. Five more tRNAs were found after the detailed manual search of non-coding regions: tRNA^Met^, tRNA^Cys^ and tRNA^Ser^ on chromosome-I, and tRNA^Asn^ and tRNA^Ile^ on chromosome-II. Whereas many metazoan genomes have clusters of five to seven tRNA genes, in monogonont rotifers, including *B*. *calyciflorus*, tRNA genes appear to be dispersed, with no clusters larger than three [9,10].

Four major NCRs notwithstanding, non-coding regions ranged from 1 to 258 bp in length in chromosome-I (13 ncrs) and from 1 to 53 bp in chromosome-II (3 ncrs). Two putative gene overlaps were found in chromosome-I (both 2 bp): tRNA^Val^—tRNA^Gly^ and *Nad2—*tRNA^Trp^; and one in chromosome-II (4 bp): *Nad4l* and *Nad4*. However, it is likely that *Nad2* used the unfinished T—stop codon instead of the complete TAA, in which case there would be no overlap with tRNA^Trp^. Regarding the *Nad4l* and *Nad4* overlap, if we consider the possibility of the unfinished T—stop codon in this sequence…ATT(ATAA)CT…, overlap could be only two bases…ATT(AT)AACT…, or there could even be no overlap at all, but in that case *Nad4l* would have to be two bases shorter:…ATT)(ATAACT… Comparable to *B*. *plicatilis*, all genes on both chromosomes would be transcribed from the same DNA strand, and all four mNCRs are in the same orientation relative to the direction of transcription. Overall, three different start codons were found: ATG (10 genes), ATA (2) and GTG (1); and two stop codons: TAA (12) and TAG (1) (Table 2). All these codons are typical for the invertebrate, and rotiferan, mitochondrial DNA [15,34]. The unfinished T—codon was not counted separately, as we presumed that it would be completed (TAA) by the posttranscriptional polyadenylation [35,36]. The orthodox initiation codon ATG was also used in 8 / 9 of the twelve protein-coding genes in *B*. *plicatilis* / *B*. *koreanus* mitogenomes, respectively [9,10]. These two species used incomplete stop codons in *Nad1*, *Nad3* and *Atp6* genes. In *B*. *calyciflorus*, it was only *Nad1* and *Nad3* (*Atp6* used TAA). ATA was the start codon for *Nad4* and *Cox3* in all three species. Hence, there appears to be a relatively high level of conservation in the start/stop codon usage among the three monogonont species.

Base composition was very similar between the two chromosomes, both of which exhibited a strong A+T bias: 68.7% in chromosome-I and 69.6% in chromosome-II. To test whether this is a consequence of the large proportion of non-coding regions, we have checked whether the base composition in mNCRs is different from the rest of the genome. A+T content was only somewhat lower in the mNCRs (66%) in comparison to the complete genome (69%), mainly due to the decrease in the amount of T: 37.5% in the genome vs. 33.0% in the mNCRs. In comparison, the base composition of the mNCRs in *B*. *plicatilis* was significantly different from the rest of the mitogenome, also due to a decrease in the amount of T in favour of other bases [9]. However, the overall A+T bias appears to be typical not only for monogonont mitogenomes (*B*. *plicatilis* = 60.7–71.7% and *B*. *koreanus* = 68.81%) [9,10], but also for syndermatan mitogenomes [15,16].

### Major non-coding regions

There were three major non-coding regions (mNCRs) in the first chromosome: mNCR-I-1 (5386 bp), mNCR-I-2 (5391 bp) and mNCR-I-3 (5369 bp); and only one in the second chromosome: mNCR-II (5441 bp). Sequences were very similar: the overall average genetic distance was only 0.02. The smallest pairwise genetic distance was found between NCR-I-2 and NCR-II (0.002), and the highest between NCR-I-2 and NCR-I-3 (0.032). All four sequences were almost identical at the 5’-end, whereas the largest differences were observed at the 3’-end; notably, a deletion of about 20–25 bases at the end of NCR-I-3 and a T-rich extension of about 46 bases at the end of NCR-II. Whereas NCRs of angiosperm mitochondria contain (usually) non-functional polypeptides that are translated from open reading frames created as by-products of genome alteration [4], we did not find putative ORFs in either of the *B*. *calyciflorus* mNCRs.

Generally, there is no correlation between the size of mitogenome and gene content, as the differences in size of mitochondrial genomes are mostly caused by marked variations in the length and organisation of intergenic regions. In *B*. *calyciflorus*, non-coding regions comprised more than half (59.75%) of the total genome: 61.09% of the chromosome-I and 56.0% of the chromosome-II. This is comparable to other extremely large metazoan mitogenomes; specifically, some molluscan mitogenomes also show transpositions of certain regions that can more than double the genome size [29,30]. The role of these regions is still debated, some are thought to be mobile and ‘selfish’, whereas others might play a role in recombination or serve as replication and transcription regulatory signals [28,37].

## Conclusions

The organisation of all three previously sequenced monogonont mitogenomes was almost identical: they had two circular chromosomes of a relatively similar size, with almost identical gene content and order (among the three species, not between the two chromosomes), and one major non-coding region on each chromosome. *B*. *calyciflorus* mitogenome is also organised into two circular chromosomes, but different in other aspects: the first chromosome possesses three mNCRs and it is almost three times larger than the second chromosome. Gene content (and order) of the two chromosomes is also quite different from other monogononts.

Even though monogonont rotifers are a model system in several research fields, very few mitogenomes have been sequenced so far. This is highly likely to be a consequence of a high plasticity of mitogenomic structure and organisation, as well as the existence of a varying number of very long, almost identical non-coding regions, which can make the PCR amplification and mitogenome sequence assembly a very difficult and time-consuming process. Publication of this sequence shall help streamline the future attempts to sequence monogonont mitogenomes, and ultimately contribute to the understanding of the evolutionary history of the unique mitogenomic organisation in this group of rotifers.
